# Spontaneous Cushing’s Disease Remission Induced by Pituitary Apoplexy

**DOI:** 10.7759/cureus.64231

**Published:** 2024-07-10

**Authors:** Jose E Esquivel, Ana B Santos, Anthony Hong, Francis Ruiz

**Affiliations:** 1 Endocrinology, Hospital San Juan de Dios, San José, CRI; 2 Medicine, University of Costa Rica, San José, CRI; 3 Endocrinology, Hospital México, San José, CRI

**Keywords:** adrenal insufficiency, spontaneous remission, pituitary microadenoma, pituitary apoplexy, cushing disease

## Abstract

Spontaneous remission of Cushing’s disease (CD) is uncommon and often attributed to pituitary tumor apoplexy. We present a case involving a 14-year-old female who exhibited clinical features of Cushing’s syndrome. Initial diagnostic tests indicated CD: elevated 24h urinary cortisol (235 µg/24h, n < 90 µg/24h), abnormal 1 mg dexamethasone overnight test (cortisol after 1 mg dex 3.4 µg/dL, n < 1.8 µg/dL), and elevated adrenocorticotropic hormone concentrations (83.5 pg/mL, n 10-60 pg/mL). A pituitary adenoma was suspected, so a nuclear MRI was performed, with findings suggestive of a pituitary microadenoma. The patient was referred for a transsphenoidal resection of the microadenoma. While waiting for surgery, the patient presented to the emergency department with a headache and clinical signs of meningism. A computed axial tomography of the central nervous system was performed, and no structural alterations were found. The symptoms subsided with analgesia. One month later, she presented again to the emergency department with clinical findings of acute adrenal insufficiency (cortisol level of 4.06 µg/dL), and she was noted to have spontaneous biochemical remission associated with the resolution of her symptoms of hypercortisolism. For that reason, spontaneous CD remission induced by pituitary apoplexy (PA) was diagnosed. The patient has been managed conservatively since the diagnosis and remains in clinical and biochemical remission until the present time, after 10 months of follow-up. There are three unique aspects of our case: the early age of onset of symptoms, the spontaneous remission of CD due to PA, which has been rarely reported in the medical literature, and the fact that the patient presented a microadenoma because there are fewer than 10 clinical case reports of PA associated with microadenoma.

## Introduction

Cushing’s disease (CD) is characterized by excessive production of adrenocorticotropic hormone by a pituitary adenoma and represents the most common cause of endogenous Cushing’s syndrome (CS) [1]. CD was first reported in 1912 by Harvey Williams Cushing, and he described 12 cases at the Peter Bent Brigham Hospital in Baltimore [2]. This disease has a global incidence of approximately 2.2 cases per 1,000,000 people and occurs more frequently in women from 20 to 50 years of age [3]. Pituitary apoplexy (PA) is a rare condition that occurs in 2-12% of cases, and it has a high morbidity and mortality rate [4]. We report an interesting case of a woman diagnosed with CD who achieved spontaneous remission of her disease after a PA.

## Case presentation

A 14-year-old female presented with a two-year history of weight gain (32 kg), depression, elevated blood pressure, type 2 diabetes mellitus, and growth failure (height less than the third percentile). Her height was 140 cm, and her BMI was 28.1 (97th percentile). At presentation, she had not yet reached menarche. Physical examination revealed Tanner 2 breast development, acne, hirsutism, moon facies, dorsocervical fat pad, central obesity, and stretch marks. Initial laboratory tests showed hemoglobin A1C of 13%, low-density lipoprotein of 167 mg/dL, triglycerides of 344 mg/dL, high-density lipoprotein of 26 mg/dL, creatinine of 0.4 mg/dL, and elevated liver enzymes. Abdominal ultrasound indicated moderate hepatic steatosis changes.

**Figure 1:**
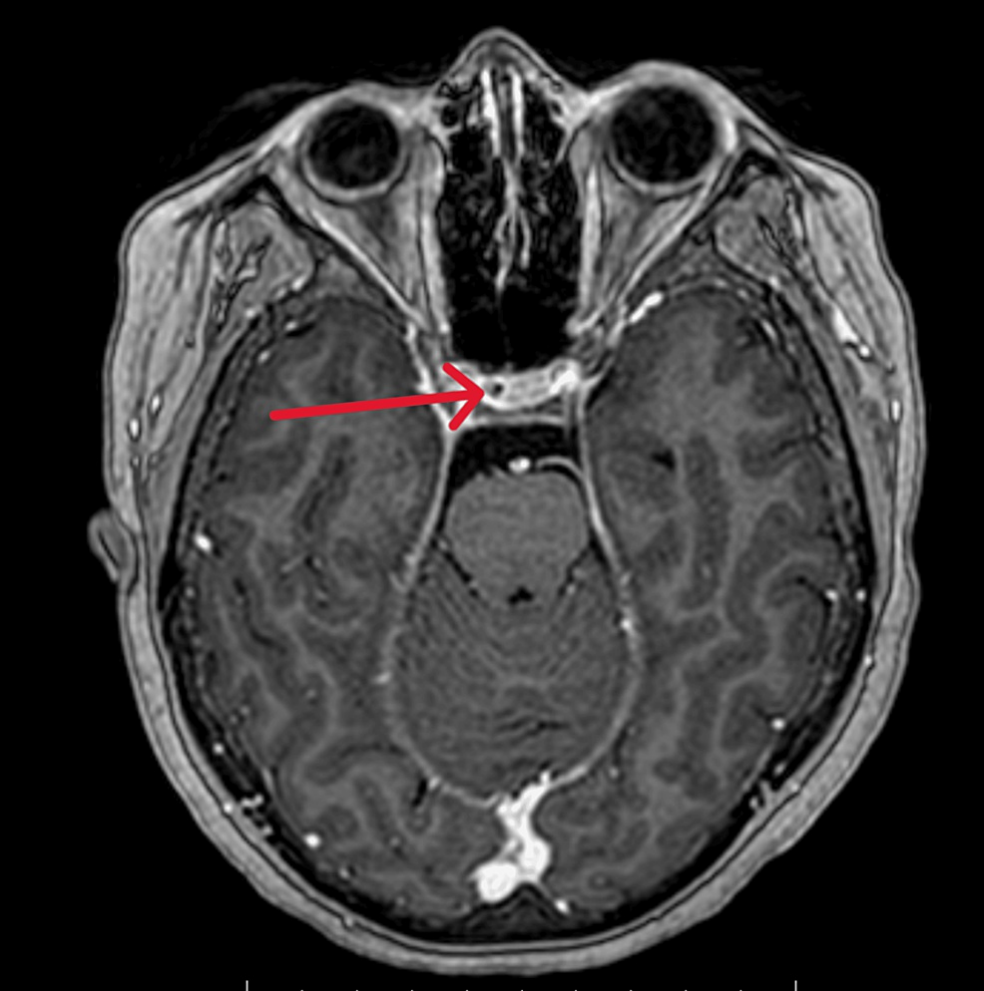
Axial view of a T1 MRI with contrast showing a sellar lesion The red arrow shows a microadenoma in relation to the normal pituitary gland.

Given the high suspicion of CS, a hormonal profile was conducted (Table 1), confirming CS and subsequently diagnosing CD. A nuclear MRI revealed a 2.6 × 1.8 mm pituitary lesion (Figure 1), prompting referral for transsphenoidal resection of the pituitary microadenoma.

**Table 1:** Pertinent laboratory investigation at baseline and follow-up with our patient ACTH, adrenocorticotropic hormone; DST, dexamethasone suppression test; IGF-1, insulin growth factor-1; TSH, thyroid-stimulating hormone

Laboratories	Reference range	Initial	One month	Three months	Six months
TSH (mUI/L)	0.35-4.94	-	2.17	-	2.01
AM cortisol (µg/dL)	6.02-18.4	17.3	4.06	<0.5	4.7
1 mg DST (µg/dL)	<1.8	3.4	-	-	-
8 mg DST (µg/dL)	<50% suppression	1.9 (78% suppression)	-	-	-
Urine-free cortisol (µg/24h)	<90	235	-	-	-
ACTH (pg/mL)	10-60	83.5	-	19.2	9.7
IGF-1 (ng/mL)	36-300	-	-	-	293

Approximately one month after the suppression tests and while awaiting surgery, the patient presented to the emergency department with a sudden, severe, holocranial headache accompanied by projectile vomiting and diplopia, suggestive of meningism. A computed axial tomography of the central nervous system was conducted, revealing no structural abnormalities. Symptoms resolved with intravenous analgesia within approximately four to six hours. Subsequently, the patient experienced a significant decrease in insulin requirements, ultimately leading to the suspension of insulin therapy due to persistent hypoglycemia.

Weeks after the headache episode, the patient was reevaluated in the emergency department with a three-day history of diffuse abdominal pain, vomiting, asthenia, myalgia, hypotension, tachycardia, orthostatism, and recurrent hypoglycemia despite insulin suspension. Acute adrenal insufficiency was suspected and confirmed by a cortisol level of 4.06 µg/dL. Treatment with intravenous hydrocortisone 50 mg every six hours was initiated, leading to complete resolution of symptoms within 72 hours. The patient was discharged on maintenance therapy with oral hydrocortisone (20 mg in the morning and 10 mg at night). Subsequent follow-ups showed undetectable cortisol levels. Currently, the patient has been followed up for 10 months post-event, showing persistent clinical and hormonal remission of her disease.

## Discussion

CD represents approximately 80% of cases of endogenous hypercortisolism, and pituitary microadenomas are the most common cause of CD in all age groups [5]. CD prevalence is 0.3-6.2 cases per 100,000 people [3], which represents 4.4% of all pituitary adenomas [6], and it is up to five times more likely to occur in women than men. Spontaneous remission of CD is rare, and it is mainly due to the apoplexy of a pituitary tumor [7].

PA is a potentially fatal condition resulting from hemorrhage or necrosis of a pituitary adenoma that produces compression of the surrounding structures with symptoms that can be critical and even fatal [8]. PA affects between 2% and 12% of patients with pituitary adenomas, mainly in nonfunctional macroadenomas [9]. Although the main mechanism of PA is hemorrhage, it can also be due to a hemorrhagic infarction or an infarction without hemorrhage; this last scenario is clinically less aggressive [10]. Among the most important precipitating factors are craniocerebral trauma, pregnancy, thrombocytopenia, coagulopathies, pituitary stimulation tests, drugs such as anticoagulants and estrogens, surgeries that are complicated by hypotension, and radiotherapy [4,11,12].

There are three unique aspects of our case. First, the age of onset is 14 years old. This characteristic has been reported in less than 6% of cases of CD, with a mean age of onset between 12.3 and 14.1 years and a slightly higher incidence in men (63%) [13]. In this population, CD is the most common cause of hypercortisolism, accounting for 75-80% of all cases [14]. Furthermore, our patient presented a significant weight gain, severe compromise in her height, hypertension, depression, and diabetes mellitus, which is compatible with the classic profile described for CD in pediatric ages. It is important to clarify that although type 2 diabetes mellitus is common in adults, it is unusual in the pediatric population [13].

Second, spontaneous remission in CD due to apoplexy has been rarely reported in the past; hence, our case is an important addition to the scant literature on this unusual phenomenon. Although there are characteristics suggestive of PA, such as hyperdense lesions within the pituitary gland and the reinforcing ring, a CT scan has a low sensitivity for detecting pituitary hemorrhage (21-46%); therefore, a negative CT scan does not rule out PA in cases where there is infarction without hemorrhage, a situation that could correspond to our case [15].

The third unique feature of our case is that the stroke occurred in the context of a microadenoma, a situation reported in less than 10 cases in the literature. Despite being a microadenoma, the symptoms of PA were severe, with symptoms of meningism, an intense headache, vomiting, and the development of adrenal insufficiency. Taylor et al. [16] reported a similar case of a 41-year-old female with microadenoma whose PA was associated with severe headache and vomiting.

The main differential diagnosis in our case is cyclical CS (CCS), a disorder that occurs in 15% of CS cases, especially in CD [17]. The diagnosis of CCS is classically established with three peaks and two valleys in cortisol secretion, spontaneous fluctuations, and clinical features of CS [7]. The possibility of CCS was ruled out due to the typical presentation of the PA event and the persistence of hypocortisolism.

Finally, several cases of recurrence of their disease have been described after remission of CS due to AP. Those recurrences usually develop in follow-ups of up to seven years [18]. At the time of the last evaluation (10 months post-PA), the patient remained in remission, but long-term follow-up is required to detect both reactivation and hypopituitarism [19].

## Conclusions

CD is a rare entity in the pediatric population, usually associated with a pituitary microadenoma. Spontaneous remission of this disease is very uncommon, but when it occurs, it is mainly due to PA. We describe a case with three unique aspects: CD with an early age of onset of symptoms, spontaneous remission of CD due to PA, which has been rarely reported in the medical literature, and the fact that there are less than 10 clinical case reports of PA associated with microadenoma. It is imperative for clinicians to be aware of this possible outcome in patients with CD.
